# Book Reviews

**Published:** 1872-08-01

**Authors:** 


### Book Reviews.

Report to the Surgeon General of the United States Army on the Minute Anatomy of Two Cases of Cancer. By Assistant Surgeon J. J. Woodward, United States Army.

This short report comprises some valuable additions to our knowledge of the histology of cancerous growths, and the two beautifully clear and distinct plates which accompany it are the finest specimens of the Photo Plate or Albertype process of printing that we have yet seen. The one representing the microscopic appearance of a section of a mammary cancer is especially accurate, and finely illustrates the immense superiority of this new process of engraving for this class of illustrations.

Spectrum Analysis Discoveries. Showing its Application in Microscopical Research and to Discoveries of the Physical Constitution and Movements of the Heavenly Bodies. Boston: Lee & Shepard. Price, 25 cents.

This little volume forms the fourth one of a series of popular science publications edited by Dana Estes. It contains numerous plates and illustrations, and its contents will be found of value to those interested in this comparatively new field of scientific investigation.

The Better Way; or, Considerations upon the Natural System of Providing for the Treatment of the Insane. By Andrew McFarland, M. D., LL.D.

(From the St. Louis *Medical and Surgical Journal.*)

An American Italy for Invalids. A Dissertation showing the Advantages, Incidents, etc., of a Journey on the Plains, in the Rocky Mountains, and Mexico, for the Cure of all Chronic Diseases. By R. E. Fullerton, M. D. H. D. Chapin & Co., 50 East Harrison street, Chicago, Publishers. Price, 25 cents.

This is a little pamphlet of fifty-four pages, and contains valuable information regarding the regions of which it treats—the accommodations, means of travel, etc.

